# Gaseous Mediators Nitric Oxide and Hydrogen Sulfide in the Mechanism of Gastrointestinal Integrity, Protection and Ulcer Healing

**DOI:** 10.3390/molecules20059099

**Published:** 2015-05-19

**Authors:** Marcin Magierowski, Katarzyna Magierowska, Slawomir Kwiecien, Tomasz Brzozowski

**Affiliations:** Department of Physiology, Jagiellonian University Medical College, Cracow 31-531, Poland; E-Mails: magierowskim@yahoo.pl (M.M.); k.jasnos@interia.pl (K.M.); skwiecien@cm-uj.krakow.pl (S.K.)

**Keywords:** nitric oxide, hydrogen sulfide, gastrointestinal tract, gastric mucosa, gastroprotection

## Abstract

Nitric oxide (NO) and hydrogen sulfide (H_2_S) are known as biological messengers; they play an important role in human organism and contribute to many physiological and pathophysiological processes. NO is produced from l-arginine by constitutive NO synthase (NOS) and inducible NOS enzymatic pathways. This gaseous mediator inhibits platelet aggregation, leukocyte adhesion and contributes to the vessel homeostasis. NO is known as a vasodilatory molecule involved in control of the gastric blood flow (GBF) and the maintenance of gastric mucosal barrier integrity in either healthy gastric mucosa or that damaged by strong irritants. Biosynthesis of H_2_S in mammals depends upon two enzymes cystathionine-β-synthase and cystathionine γ-lyase. This gaseous mediator, similarly to NO and carbon monoxide, is involved in neuromodulation, vascular contractility and anti-inflammatory activities. For decades, H_2_S has been known to inhibit cytochrome c oxidase and reduce cell energy production. Nowadays it is generally considered to act through vascular smooth muscle ATP-dependent K^+^ channels, interacting with intracellular transcription factors and promote sulfhydration of protein cysteine moieties within the cell, but the mechanism of potential gastroprotective and ulcer healing properties of H_2_S has not been fully explained. The aim of this review is to compare current results of the studies concerning the role of H_2_S and NO in gastric mucosa protection and outline areas that may pose new opportunities for further development of novel therapeutic targets.

## 1. Introduction

Gastric mucosa is constantly exposed to exogenous food products, providing vital nutrients to the human body in order to maintain physiological homeostasis. Unfortunately, many of these food products, harsh substances and drugs delivered via the oral route can affect gastric mucosal integrity. Ethanol, nicotine and ingestion of drugs, in particular, nonsteroidal anti-inflammatory drugs (NSAIDs) (e.g., aspirin, ASA) are considered as the major causative factors in the development of acute mucosal damage and gastric ulcers [1,2,3]. Moreover, *Helicbacter pylori* infection, hyperosmolar solutions, bile salts, the exposure to chronic stress, and ischemia to the gastric tissue followed by reperfusion were all reported to act as the risk factors of peptic ulcer disease [4,5].

**Figure 1:**
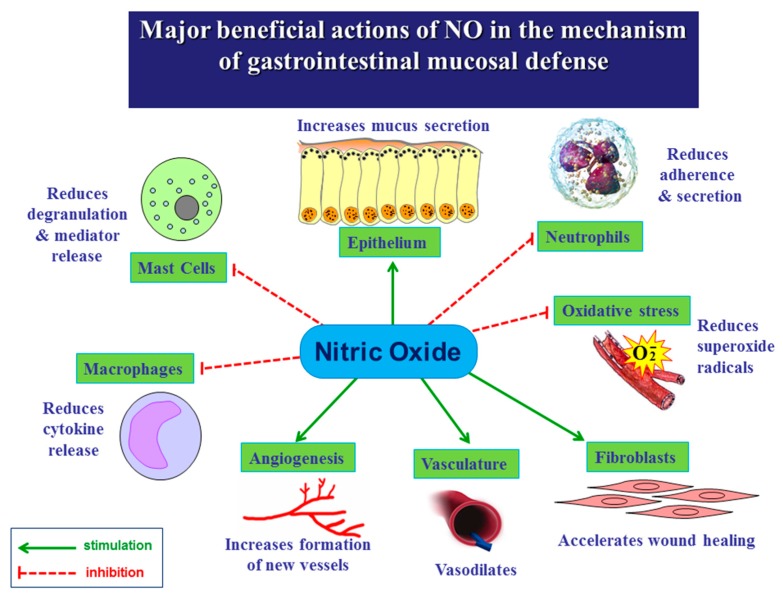
Beneficial actions of nitric oxide (NO) in the mechanism of gastrointestinal mucosal defense.

The physiological protective mechanisms involved in maintaining gastric mucosa integrity include epithelial cells secreting mucus and bicarbonate, the gastric blood flow (GBF) [6,7], endogenous prostaglandins (PGs) [8,9,10,11], metallothionein [12], melatonin [13] and recently discovered food intake controlling peptides such as ghrelin [14], orexin-A [15] and leptin [16]. Moreover, gaseous molecule nitric oxide (NO) and other gaseous vasoactive mediators such as hydrogen sulfide (H_2_S) and carbon monoxide (CO) were shown to play an important role in the mechanism of mucosal defense and gastroprotection [17,18]. It is now generally accepted that gaseous mediators NO (Figure 1) and H_2_S contribute to many physiological and pathophysiological processes including the maintenance of gastrointestinal (GI) integrity and the mechanism of gastroduodenal protection.

## 2. Biosynthesis of NO and Its Major Functions in Various Body Systems

NO is produced and released from vascular endothelium and sensory nerve endings via the enzymatic activity of constitutive NO synthase (cNOS) and inducible NOS (iNOS) [19]. The agonists, such as acetylocholine (ACh), bradykinin or serotonin (5-HT) were shown to stimulate their membrane receptors in endothelial cells of gastric vessels and release NO [19]. A substrate for NO synthase to produce NO is amino acid l-arginine [20]. NO diffuses from endothelium to smooth muscles, located in vascular wall, where NO reacts with soluble guanylyl cyclase (sGC), leading to cellular rise of a second messenger cyclic guanosine monophosphate (cGMP). NO activates sGC, transforming guanosine triphosphate (GTP) to cGMP. This cGMP, acting via protein kinase G leads to relaxation of smooth muscle cell and subsequent increase of vessel diameter and an enhancement in the organ blood flow [8,21]. The biological action of NO may be mimic by the exogenous administration of NO donors, such as sodium nitrate, nitroprusside or other organic nitrates, the 3-morpholinosydnonimine (SIN-1), *S*-nitroso-*N*-acethyl-d,l-penicylamine (SNAP), gliceryl trinitrate (GTN) or NO-releasing aspirin [22,23]. Thiols (R-SH), for example, glutathione (GSH) cooperate with NO. Other vasodilators, for example, pentoxifylline (PTX) may act on smooth muscle causing their relaxation but this effect seems to be NO independent [24].

The fact that NO acts on blood vessels causing vasodilatation implies that this gaseous molecule contributes to the maintenance of gastric mucosal barrier integrity. This is supported by the observation that the inhibition of NO production by a nonspecific N^G^-nitro-l-arginine (l-NNA) not only markedly impaired gastric secretion and gastric motility but also abolished the protective activity of gastroprotective agents [25]. Moreover, the inhibition of NOS has been shown to delay the healing of chronic gastric ulcers and diminish the restoration of the GBF at ulcer margin associated with this healing process [26]. Interestingly, the adverse effect of blockade of NOS by l-NNA or l-N^G^-monomethyl arginine citrate (l-NMMA) on gastric integrity can be reversed by administration of l-arginine, a substrate for this enzyme, administered in the presence of this inhibitor [21,26].

## 3. Role of NO in the Mechanism of Gastric Integrity, Protection and Ulcer Healing

The evidence based medicine indicate that NO, the afferent capsaicin-sensitive C fibers and products of cyclooxygenase (COX) activity, are major factors, involved in the maintenance of gastric mucosal integrity due to their potent role in the control of the GBF, gastroprotection and ulcer healing [21,26].

Inhibition of NOS that results in a decrease in local NO production, impairs gastric microcirculation and aggravates gastric lesions induced by noxious agents. In physiological conditions, NO is produced by NOS from l-arginine, which is transformed to l-citrulline [27]. In pathological conditions, l-arginine may be involved in another metabolic pathway, catalyzed by protein arginine methyltransferase (PRMT). PRMT, in presence of proteins containing methylated arginine residues, produces asymmetric dimethylarginine (ADMA) [28]. ADMA acts as an endogenous NOS inhibitor in decreasing the NO production. Depletion of NO has multifactorial consequences and may be considered as a phenomenon in pathogenesis of numerous global diseases such as hypertension, arteriosclerosis, heart failure, chronic kidney disease, diabetes mellitus [29,30,31,32]. Then, ADMA can be metabolized to l-citrulline by enzyme dimethylarginine dimethylaminohydrolase (DDAH) [32]. Recently, the administration of ADMA failed to cause spontaneous gastric lesions but exacerbated gastric lesions induced by various ulcerogenes including stress and ischemia-reperfusion injury [33,34] suggesting that the inhibition of NO-synthase can increase the gastric mucosa susceptibility to damage induced by various stressors and gastric ulcerogenes.

Mechanism of beneficial action of NO-donors has been attributed to the vasodilatation induced by these agents resulting in an increase of perfusion of target organs [35]. For instance, SIN-1-induced release of NO exerted anti-atherogenic properties by alteration of low density lipoprotein (LDL) metabolism in macrophages [36]. Another NO-donor administration, *S*-nitroso-*N*-acethyl-d,l-penicylamine (SNAP), potently affected the regulation of cardiovascular system in hypertension [37]. The administration of NO-donors accelerated healing of gastric mucosa damage and experimental gastric ulcers [38]. For example, GTN attenuated damaging effect of ethanol by improvement of the changes in potential difference across the stomach wall [39]. SNAP demonstrated gastroprotective properties against ethanol-induced gastric lesions due to an increase in the GBF in this animals. Moreover, SNAP has been shown to inhibit gastric acid secretion documented *in vitro* in isolated parietal cells, which at least in part, may contribute to the observed gastric protection by this agent *in vivo* [40].

The major complication related to NSAIDs such as ASA ingestion in humans is the increased risk of adverse GI-side effects associated with their world-wide use as anti-inflammatory therapy. These adverse effects of ASA were originally attributed to the inhibition of COX and the deficiency of endogenous PGs, an increase in reactive oxygen species (ROS), lipid peroxidation and a fall in antioxidizing activities of gastric mucosa exposed to ASA [41]. The mechanism of NSAID-induced side effects is inhibition of constitutive isoform COX-1 and inducible isoform COX-2 [41]. The COX-1 plays gastroprotective role, because it produces PGs involved in protection of GI-mucosa while COX-2, which is induced but proinflammatory mediators, results in detrimental effects such as an increase of vessels permeability, pain and fever due to production of large amount of proinflammatory PGs [42,43]. The administration of non-selective COX inhibitors, e.g., ASA causes, except for therapeutic effects resulting from COX-2 inhibition, also side effects, resulting from COX-1 inhibition [42]. However, the selective inhibition of COX-2 aggravates acute gastric lesions induced by stress and ischemia-reperfusion and delays the healing of preexisting gastric ulcers [43].

A new class of NO-releasing NSAIDs was shown to inhibit COX-1 and COX-2 activity and PGE_2_ generation without causing mucosal damage [22,44]. Furthermore, NO-ASA, despite inhibition of COX enzymes was shown to protect the gastric mucosa against ethanol, stress and NSAID-induced gastric damage and accelerate the healing of gastric ulcers, mainly due to release of NO enhancing GBF [44,45,46]. Mechanism of this beneficial action of NO should be further investigated but NO, which is released from NO-ASA, could compensate for the inhibition of COX-1 and COX-2 activity and subsequent fall in PG synthesis induced by ASA [47]. Fiorucci *et al*. [48] demonstrated that NO-ASA compared with native ASA exerted sparing effect on gastric mucosa by inhibition of apoptosis and impairment of proinflammatory cytokines TNF-α and IL-1β. Takeuchi *et al*. [49] have confirmed the protective activity of NO donating ASA against formation of gastric lesions induced by cold stress. However, the excessive release of NO from its donors could exert deleterious influence on the gastric mucosa because the application of SNAP in higher doses exaggerated ethanol-induced gastric damage [40].

The scavenging effect of these new NO derivatives of NSAID on ROS production has been demonstrated during healing of chronic gastric ulcers and this action was accompanied by a decrease in lipid peroxidation [50]. Wallace *et al*. [51] revealed that NO-ASA exhibited inhibitory effect on neutrophil adherence to the vascular endothelium and the neutrophil infiltration of gastric tissue and these effects resulted in diminishing of oxidative GI tissue damage. Neutrophils produce superoxide radical anion (O_2_^•**−**^), which belongs to group of reactive oxygen species (ROS). Superoxide radical anion reacts with cellular lipids, leading to the formation of lipid peroxides. The major anti-oxidative enzyme is superoxide dismutase (SOD). SOD catalyzes the dismutation of superoxide radical anion (O_2_^•**−**^) into less noxious hydrogen peroxide (H_2_O_2_), that is further degraded by catalase or glutathione peroxidase (GPx) [52,53]. The reaction of superoxide (O_2_^•**−**^) with NO is however approximately three times faster than the elimination of superoxide by SOD, which could question the role of SOD in protecting NO bioavailability. Animal models have however clearly indicated that the inhibition of SOD by diethyldithiocarbamate (DETC) leads to a very significant attenuation of endothelium dependent NO-mediated vasorelaxation [53].

In contrast, Konaka *et al*. [54] observed an intensification of lipid peroxidation and myeloperoxidase (MPO) activity, accompanied by increase of NO production in rats with indomethacin-induced small intestinal lesions. The alternative therapy against GI lesions induced by ASA could be administration of other NO-donors because the experimental combined therapy of ASA with GTN, SIN-1, SNAP, molsidomine, sodium nitroprusside ameliorated the formation of ASA-induced gastric damage and improved the status of gastric mucosa treated with non-selective and selective COX-1and COX-2-inhibitors [52,53,55].

Disturbances in blood perfusion of gastric mucosa, during stress, result in local episodes of ischemia to the gastric tissue, followed by reperfusion and enhanced generation of ROS [56]. Previous studies revealed that ROS may cause a peroxidation of membrane lipids to lipid peroxides and impairment of cellular physiological functions leading to increased acid back-diffusion [56,57]. The determination of SOD activity and level of reduced glutathione (GSH) serve as suitable for the assessment of antioxidizing status of gastric mucosa injured by various damaging agents [57,58]. Three types of SOD can be distinguished: cytoplasmatic, mitochondrial and extracellular. SOD catalyzes the dismutation of superoxide radical anion (O_2_^•**−**^) into less noxious hydrogen peroxide (H_2_O_2_), that is further degraded by catalase or glutathione peroxidase. Catalase is an enzyme which accelerates degradation of H_2_O_2_ into water and oxygen. The second pathway of H_2_O_2_ metabolism depend on activity of GPx and cooperating glutathione reductase. The reduction of H_2_O_2_ into water by GPx is accompanied by the conversion of GSH into oxidized form (GSSG) [58]. Interestingly, NO-releasing ASA increased gastric mucosal expression of anti-oxidative enzymes SOD and GPx in rats with stress-induced gastric lesions and greatly attenuated the rise in mucosal expression and release of proinflammatory cytokines IL-1β and TNF-α [58].

## 4. Role of NO in the Esophageal and Intestinal Protection

The esophageal mucosal integrity depends upon the non-keratinized stratified squamous epithelium, hydrophobic lipid bilayer, tight junctions and intensive cell replication and regeneration after acid exposing mainly the distal esophageal mucosa [59]. Importantly, the esophagus, unlike stomach and duodenum, has no viscoelastic surface mucous layer and its epithelial cells do not secrete bicarbonate [59,60,61]. Consequently, the esophagus does not effectively trap of luminal bicarbonate allowing for a buffering gastric acid as it back diffuses from lumen toward epithelium [62]. To keep such acidity from injuring the cell, cell membrane is capable of removing excess H^+^ from the cell and restoring pH to neutrality due to a sodium-dependent, chloride-bicarbonate, exchanger and a sodium-hydrogen ion exchanger of isotype-1 [63,64]. In esophageal epithelial cells, these transporters are localized to the basolateral membrane and include a sodium-dependent, chloride-bicarbonate, exchanger and a sodium-hydrogen ion exchanger of isotype-1 [64,65]. These barrier and transport functions of the esophageal epithelium as well as the adequate blood supply are integral processes for protection of the tissue against injury upon exposure to gastric acid [63,64,65,66].

NO and guanylate cyclase signaling were proposed to play a major role in the control of lower esophageal sphincter (LES) relaxation after stimulation of intrinsic inhibitory motor neurons [62]. In studies by Lanas *et al*. [67,68], the preexposure of the esophageal mucosa to acidified saline significantly decreased both the mucosal damage and the mucosal barrier dysfunction induced by acidified pepsin. The concomitant treatment with either the nitric oxide synthase inhibitor, L-NNA or the perfusion of immunospecific EGF-receptor antibodies or tyrphostin-25, an inhibitor of the tyrosine kinase activities [67,68] completely reversed the protection induced by acid. They concluded that the rabbit esophageal mucosa develops mucosal adaptation to acid and pepsin dependent, at least in part, on nitric oxide and EGF-receptor-mediated mechanisms [67,68]. NO could mediate the esophagoprotective activity of certain radical scavenging substances e.g., melatonin, because the administration of l-tryptophan, a precursor of this indoleamine, or exogenous melatonin itself attenuated the esophageal damage in experimental models of rodent esophagitis [69,70]. Moreover, angiotensin-(1-7), a major vasoactive metabolite of angiotensin I, prevented the esophageal damage induced by experimental reflux esophagitis in rats *via* the modulation NO/NOS activity and gastric epithelial NO release [71]. In contrast, the co-administration of sodium nitrite and ascorbic acid in study by Ishiyama *et al*. [72] aggravated the esophageal damage compared with baseline reflux esophagitis, while the damage was unchanged when either of the reagents alone was given. This aggravatory effect of NO has been referred to the diffusion of the luminal NO into the adjacent superoxide-enriched inflamed tissue of the esophagus and excessive production of the highly toxic agent peroxynitrite, thus causing exacerbation of esophageal damage [72]. This notion was supported in their study [72] by observation that superoxide scavengers efficiently prevented the exacerbation of esophageal damage by exogenous NO exposure, suggesting an essential role of superoxide in the development of esophageal injury induced by gastric reflux. Interestingly, variations in saliva nitrite concentration in swallowed saliva failed to modify LES pressure and rate of gastric emptying and did not predispose to gastro-esophageal reflux (GERD) symptoms in humans [73]. The conventional NSAID such as ASA have been shown to augment esophagitis in experimental animals and humans but the new NSAID-releasing NO such as NO-ASA, exerted the beneficial protective effect against reflux esophagitis via the enhancement of esophageal microcirculation due to NO release and an inhibitory effect of this gaseous molecule on expression and release of pro-inflammatory cytokines [74].

Both ulcerative colitis and Crohn’s disease are collectively included in the chronic intestinal disorders of inflammatory bowel diseases (IBDs) which reflect a chronic and relapsing inflammatory condition of the GI-tract [75]. The pathophysiology of IBD involve the combination of factors including patients’ genetic predisposition, immune dysregulation, barrier dysfunction and the alterations in microbiota [75]. The environmental factors, such as changes in diet, stress, the use of antibiotics, smoking or improved domestic hygiene (e.g., eradication of intestinal helminthes) were implicated in the development and an increased prevalence of IBD world-wide [76]. The role of NO in IBD is controversial since there is evidence of proinflammatory and beneficial actions of this endogenous and exogenous gaseous molecule in the treatment of IBD [77,78]. For instance, a protective action of exogenous NO in inflammation has been suggested by demonstration the treatment with *Lactobacillus farciminis*, which produces NO *in vitro* attenuated the colonic damage in experimental TNBS-induced colitis with the extent similar to that exhibited by sodium nitroprusside (SNP), a NO donor [77]. In another study, the increased production of pro-inflammatory cytokines and NO through the inducible nitric oxide synthase (iNOS) pathway has been proposed to play a role in pathogenesis of human ulcerative colitis (UC) [78]. In their study [78], the inflamed and not inflamed mucosa from patients with severe UC were incubated with a highly selective iNOS inhibitor 1400W, with or without a relatively selective cNOS inhibitor l-NAME, or an NO-donor, SNAP. They concluded that NO seems to exacerbate the inflammatory response, and selective iNOS inhibition may have therapeutic promise in the treatment of UC [78]. This notion was supported by observation that the selective inhibition of iNOS besides inhibition of NO release, suppressed mucosal TNF-α and IL-6 release in colonic mucosal explants of patients with active UC [78]. The protective role of NO in rodent models of experimental dextran (DSS)-induced colitis was developed by Jädert *et al*. [79], who revealed that inorganic nitrate and nitrite can serve as an alternative substrates for NO generation in the GI-tract. The administration of nitrate in their study [79] inhibited the disease activity score (DAI) and improved DSS-induced colitis by increasing the thickness of the protective mucus secretion in colonic mucosa. Nitrite not only alleviated inflammation associated with DSS administration but also displayed therapeutic effects by ameliorating established colonic inflammation due to the attenuation of enhanced colonic expression of iNOS and the preservation of adherent mucus layer [79]. Arginase is the endogenous inhibitor of inducible NO synthase (iNOS), that uses the same substrate, l-arginine and synthesizes ornithine, which is metabolized by the enzyme ornithine decarboxylase (ODC) to produce polyamines [80]. In a study using the same animal model of DSS-induced colitis, the administration of nor-NOHA, an inhibitor of arginase activity, ameliorated the colonic damage and the upregulation of arginase at both mRNA and protein levels, and decreased the content of l-arginine in colonic tissue [80]. As a result, the decreased concentration of NO_x_ in colonic tissues during colitis was restored to almost normal levels [80]. These authors concluded that arginase-induced depletion of NO production could contribute to the pathogenesis of the colonic inflammation and arginase inhibition should be further considered as the therapeutic strategy in the treatment of colitis [80].The dysbiosis of bacteria as a source of NO has been hypothesized to initiate UC in humans and the prolonged production of bacterial NO with sulphide could contribute to the initiation and mucosal barrier breakdown [81]. It is proposed that the production of NO by colonic bacteria and that produced by the colonic mucosa should be considered as two separate sources of NO in the lumen of lower GI-tract [81].

## 5. Biosynthesis of H_2_S and Its Major Functions in Various Body Systems

**Figure 2:**
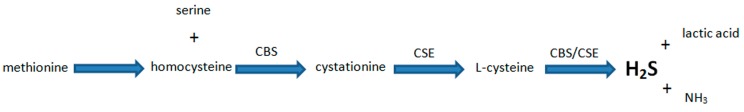
Simplified hydrogen sulfide synthesis pathway.

H_2_S is biosynthesized form l-cysteine by the activity of two main pirydoxal-5-phosphate (vitamin B_6_) dependent enzymes: cystathionine γ-lyase (CSE) and cystathionine β-synthase (CBS) (Figure 2) [82,83]. Moreover, H_2_S may be synthesized by 3-mercaptopyruvate sulfotransferase (3-MST) in coactivity with cysteine aminotransferase [84,85]. H_2_S concentration in mammalian blood ranges between 30 and 100 µM. In the brain upper limit is 160 µM. Values higher than 200 µM exerts toxicity [86,87].

In experimental models, d,l-propargylglycine (PAG) or β-cyanoalanine are commonly used to inhibit CSE activity, whereas hydroxylamine or aminooxyacetic acid serve as a tool to inhibit CBS [88,89]. To increase endogenous H_2_S level, researchers use precursor of the gaseous mediator synthesis, l-cysteine or a direct donors of this gaseous mediator, such as NaHS, diallyl disulfide (DADS) or Lawesson’s reagent [2,90].

Recent studies have shown that H_2_S, likewise NO and CO, is involved in various physiological activities [17,87]. Those gaseous molecules indicate vasodilatory, neuromodulatory and anti-inflammatory effects [91,92].

In the central nervous system, H_2_S dose-dependently evokes long-term potentiation and this response is accompanied with intracellular cAMP production and NMDA-receptors activity in hippocampus [92]. Additionally, H_2_S protects blood-brain barrier integrity [93] and promotes angiogenesis after cerebral ischemia [94].

In the cardiovascular system H_2_S inhibits leukocytes adherence to blood vessels walls and induces vasodilatation [95]. Both abovementioned effects were abolished by glibenclamide, which suggests that H_2_S physiological actions are connected with ATP-dependent potassium ion channels [96]. Interestingly, the hypotensive effect of NO and CO is linked to sGC and cGMP activation what differentiates those molecules from H_2_S [97].

Many studies have been conducted to determine the role of H_2_S in the treatment of inflammatory pathologies [98,99]. On the one hand, a pro-inflammatory action of H_2_S was observed in lipopolysaccharide (LPS)-induced endotoxemia [100,101] and therapeutic administration of PAG, H_2_S-synthetizing enzyme inhibitor, has been shown to protect mice against acute pancreatitis associated lung injury [102]. On the other hand, many studies reported anti-inflammatory features of H_2_S [99]. The ability of H_2_S to reduce inflammation has been demonstrated in a variety of animal models, including mice burn injury [103], rat model of colitis [104] and carrageenan-induced paw edema [105]. H_2_S can evoke anti-inflammatory and pro-inflammatory effects depending on lower and higher concentration, respectively [95,105].

In the GI system H_2_S remains important in the regulation of local homeostasis and may be physiological factor with an essential role in gastric mucosal defense mechanisms.

## 6. Involvement of H_2_S in the Mechanism of Gastroprotection and Ulcer Healing

It has been demonstrated that H_2_S protects GI tract against gastric damage induced by various factors [89]. Wallace *et al*. [106] showed that COXs inhibitors in combination with H_2_S donor can attenuate gastric mucosal lesions in stomach induced by NSAID, such as naproxen or ASA. It has been shown that H_2_S releasing derivatives of NSAID have reduced side effects within GI tract comparing with native forms of this agents [107]. These results were confirmed by Liu *et al*. [108] by demonstration that the novel H_2_S-releasing derivative of this anti-inflammatory drug attenuated gastric lesions induced by conventional ASA.

Cipriani *et al*. [109] demonstrated that protective action of H_2_S is accompanied with the activity of bile acid receptor (GPBAR1) since GPBAR1 agonists protected gastric mucosa against injury induced by ASA and other NSAIDs in a COX-independent manner. Administration of PAG reversed these effects. It was concluded that GPBAR1 and H_2_S are essential to maintain GI tract integrity against exposure to damaging factors [109].

Lou *et al*. [110] demonstrated that H_2_S decreases the concentration of the lipid peroxidation products in gastric mucosa and average gastric mucosa lesion number in the animal model of the water immersion and restraint stress. Our recent study revealed that endogenous PGs and afferent sensory nerves are involved in gastroprotective effect of H_2_S against stress-induced gastric damage [111]. Moreover, H_2_S dose-dependently exerted protective activity within the rat gastric mucosal cells (RGM1) against H_2_O_2_-induced oxidative damage. Involvement of mitogen-activated kinases (MAP) in H_2_S gastroprotection was observed. After inhibition of c-Jun *N*-terminal kinases (JNK) activated by oxidative stress and involved in apoptosis process, protective effect of NaHS was abolished. JNK-dependent intracellular signaling pathway is involved in protective action of H_2_S released from NaHS, although, a relative effect was not observed during inhibition of particular isoforms of p38 proteins which have similar function to JNK [112]. Taken together, H_2_S exerts anti-oxidative activity and plays an important role in protection of gastric mucosa epithelial cells against oxidative stress via MAP kinases activation.

**Figure 3:**
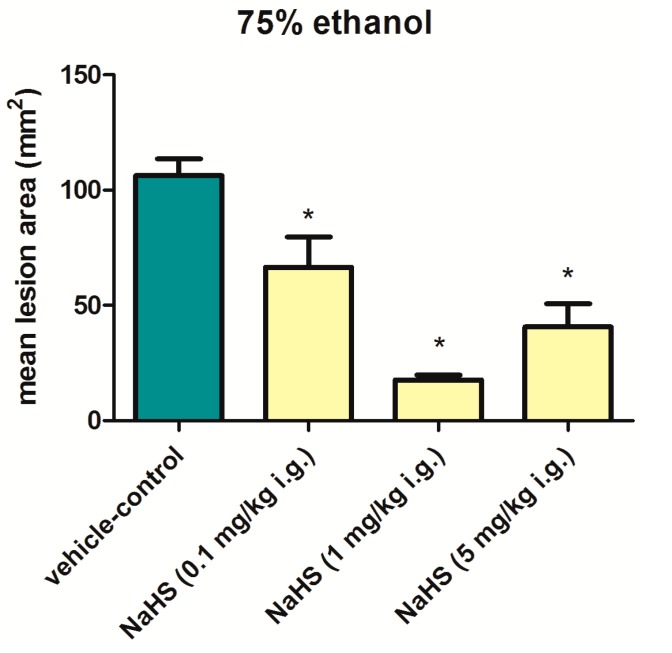
Mean lesions area of rats’ gastric mucosa exposed to 75% ethanol. Thirty minutes before intragastric (i.g.) application of 75% ethanol, animals were pretreated with saline or NaHS (0.1–5 mg/kg). Results are mean ±S.E.M of 6 rats per each group. Asterisk indicates significant (*p <* 0.05) difference in mean lesions area as compared with vehicle-control group.

In our own study, we observed that NaHS, the donor of H_2_S, administered intragastrically in graded doses ranging from 0.1 mg/kg up to 5 mg/kg 30 min before application of 75% ethanol significantly reduced the area of gastric damage with the highest dose of 5 mg/kg being not more effective than 1 mg/kg of this donor (Figure 3). Interestingly, Chávez-Piña *et al*. [3] demonstrated that PAG reduced ethanol-induced gastric lesions area comparing to NaHS-treated group and saline treated control groups with and without exposure to ethanol. Increased concentration of H_2_S was measured in gastric mucosa of rats exposed to ethanol comparing with saline treated animals. Inhibition of CSE by PAG resulted in decrease of gastric lesions area and H_2_S concentration. It is worth emphasizing that indomethacin inhibited the effect of PAG suggesting that PAG-induced gastroprotection could depend upon the generation of endogenous PGs [3]. We assume that there is a threshold level of H_2_S concentration in the tissue which determines if this gaseous molecule exerts beneficial or harmful effects in gastric mucosa. Further studies are certainly required to prove the role of this endogenous gaseous molecule in gastroprotection against necrotizing types of injury induced by ethanol.

The undisturbed GBF is very important in homeostasis of the GI tract. As mentioned above in this review, the dysfunction of GBF may lead to initiation of gastric perturbations including mucosal lesions. Kubo *et al.* [113] observed that exposure to high and low concentrations of NaHS induced vasorelaxation and vasoconstriction, respectively, in isolated gastric blood vessels. Moreover, the contractile effect was accompanied by the inhibition of NOS and the endothelium-derived hyperpolarizing factor (EDHF). The relaxation of the blood vessels was partially inhibited by glibenclamide administration. Additionally, intravenous injection of NaHS increased GBF in rats [113]. We can assume that with partial involvement of ATP-activated calcium ion channels H_2_S takes part in relaxation of blood vessels in GI tract [113].

It was shown that l-cysteine and NaHS exert gastroprotection in animal model of ischemia/reperfusion-induced gastric injury [114]. The mechanism was accompanied by the decrease of mRNA expression for pro-inflammatory cytokines, such as IL-10 or TGF-β in blood samples observed after application of H_2_S precursor and donor while PAG reversed this effect [114]. NaHS also prevented I/R-induced oxidative stress and inflammation by decrease in MDA content, increase in GSH level and decrease of NO, IL-6 and TNF-α secretion in gastric mucosa [115]. H_2_S significantly attenuated p38 and JNK proteins activity stimulated after exposure of gastric mucosa to ischemia and reperfusion [115]. It was shown that H_2_S exerts antioxidant effect via Keap1 s-sulfhydration induced Keap1/Nrf2 disassociation and Nrf2 activation [115]. l-cysteine protected gastric mucosa against ischemia-reperfusion injury by enhancing the anti-oxidative capacity of the tissue through increasing GSH and SOD levels [116].

It has been shown that NaHS and l-cysteine reduced distention-induced gastric acid secretion while l-NAME, an inhibitor of NO biosynthesis reduced this effect, which suggests the involvement of NO in mediating the antisecretory effect of H_2_S [117]. However, Takeuchi *et al*. [118] reported that H_2_S released from NaHS increased HCO_3_^−^ secretion in the stomach. This effect was mediated by capsaicin-sensitive afferent neurons and dependent on NO and PGs, but not by ATP-sensitive K^+^ channels. Nicolau *et al*. [119] demonstrated that Lawesson’s reagent, H_2_S donor protects gastric mucosa against alendronate-induced gastric damage by reduced lipid peroxidation as confirmed by a decrease in malonyldialdehyde (MDA) formation and MPO activity, increased GSH level, and reduced concentration of TNF-α and IL-1β in gastric tissue. Interestingly, in this case, glibenclamide reversed beneficial effect of Lawesson’s reagent suggesting involvement of K^+^-ATP channels activity in H_2_S gastroprotection against alendronate-induced injury.

**Figure 4:**
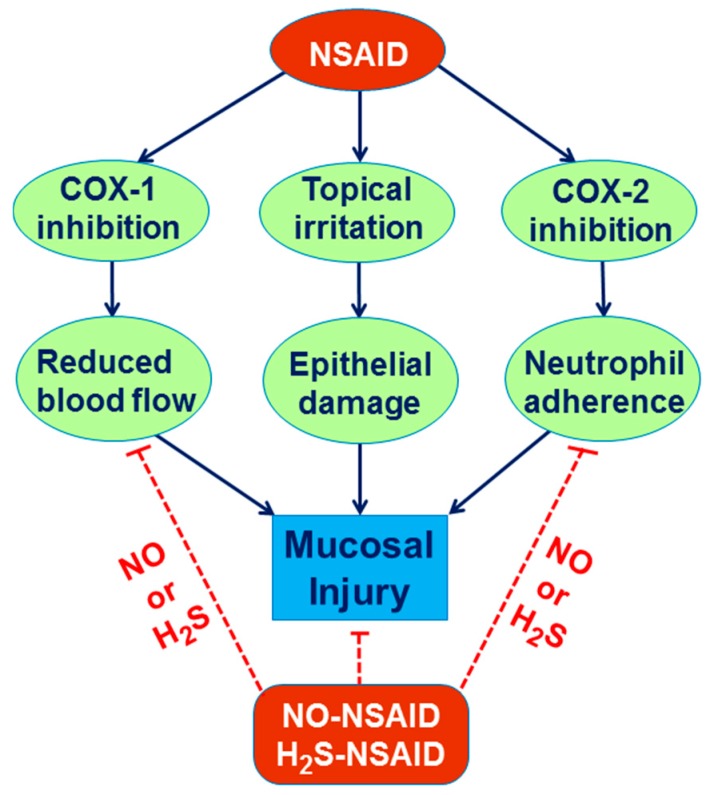
NO and H_2_S gastroprotection against NSAID-induced gastric damage.

The therapeutic efficacy of H_2_S was taken into the consideration because H_2_S-releasing derivatives of drugs were developed. It has been shown that H_2_S-releasing aspirin (ACS14) caused reduced gastric damage as compared with standard form of the drug [108]. Wallace *et al*. [120] demonstrated that H_2_S-releasing derivative of naproxen (ATB-346) exhibits anti-inflammatory properties similar to naproxen while gastrointestinal toxicity of this agent is reduced. Moreover, Wallace *et al.* [90] demonstrated that Lawesson’s reagent administration accelerated gastric ulcer healing in experimental model. Taken together, gastroprotective activity of H_2_S combined with NSAIDs could be an attractive option in the future as the new alternative anti-inflammatory therapy in human subjects taking native NSAIDs with complicated upper and lower GI disorders (Figure 4).

## 7. Role of H_2_S in the Esophageal and Intestinal Protection

Wallace *et al*. [121] have suggested that enteric bacteria may be main source of H_2_S in GI tract. The molecule can be alternative for oxygen for mitochondrial respiration. Diffusion of H_2_S released from bacteria into the subepithelial area is controlled by enterocytes and colonocytes. This interaction could be important to modulate mucosal function and integrity. H_2_S was shown to inhibit spontaneous contractile activity of smooth muscle cells in rat stomach and jejunum [122].

H_2_S evokes Cl^−^ ion secretion by activating Ca^2+^ and ATP-sensitive K^+^ channels, what was demonstrated in rat colon [123]. Moreover, it has been shown that NaHS dose-dependently increases HCO_3_^−^ ion secretion in rat’s small intestine. This ions are important compound of natural protective alkaline mucus neutralizing acidic gastric contents which enters the duodenum from the stomach. Ise *et al*., demonstrated that NaHS increased bicarbonate secretion after HCl (10 mmol/L) infusion into rat duodenum. Glibenclamide did not inhibited this effect what confirms that the mechanism may not depend on potassium channels activity. PAG decreased secretion of protective bicarbonates as compared to control group [124].

It has been shown that H_2_S protected the small intestine against dextran sodium sulfate (DSS)-induced colitis in mice [125]. Administration of PAG significantly increased the intestinal damage score observed as a bleeding, changes in a stool consistency and a weight loss. This effect was accompanied by the neutrophil activation observed as the increased MPO activity while H_2_S donors reduced this effect [125]. H_2_S exerted cytoprotection against trinitrobenzenesulfonic acid (TNBS)-induced colonic damage via excitation of sensory nerves and activation of Ca(v)3.2 T-type Ca^2+^ channels [126]. Moreover, H_2_S donors such as allyl sulfides from garlic reduced the severity of the colitis in experimental models [127,128,129].

Flannigan *et al*. [130] demonstrated that hyperhomocysteinemia exacerbated colitis what was accompanied by decreased colonic H_2_S synthesis cross-regulated by IL-10 production in colonic tissue suggesting that the IL-10/H_2_S signaling pathway could be promising target in therapy of inflammatory bowel disease.

We cannot exclude potent therapeutic value of H_2_S against e.g., gastroesophageal reflux disease (GERD) and Barrett’s disease since Zayachkivska *et al*. [131] have demonstrated that H_2_S serves as a protective factor against non-erosive esophagitis The protective effect of H_2_S against esophagitis and GERD in humans await further experimental and clinical studies.

## 8. Experimental Section

The study was approved by the Institutional Animal Care and Use Committee of Jagiellonian University Medical College in Cracow (Country) and run in accordance with the statements of the Helsinki Declaration regarding handling of experimental animals. Male Wistar rats with weight averaging about 250 g were used in this study. Animals were fasted for 24 h with free access to drinking water before the experiment. 30 min before application of 75% ethanol, rats were randomly selected into the groups and were pretreated i.g. with: (1) vehicle (saline; 1 mL/rat) and (2) NaHS applied in graded doses ranging from 0.1 mg/kg up to 5 mg/kg. One hour after application of 75% ethanol, animals were anesthetized with pentobarbital (60 mg/kg i.p.) and sacrificed by cervical dislocation, the abdomen was opened and the stomach was removed to determine the area of gastric lesions by computerized planimetry (Morphomat, Carl Zeiss, Berlin, Germany) by the person who did not know to whom experimental group animals belonged to [7,33].

## 9. Conclusions

**Figure 5:**
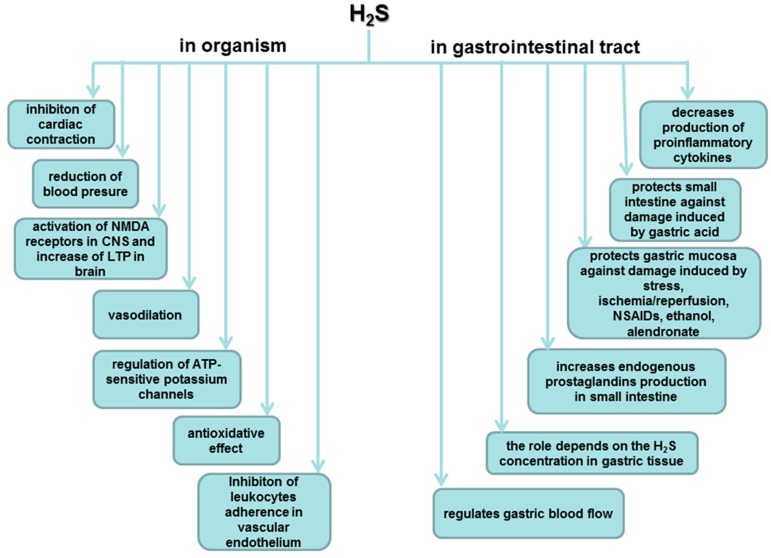
Role of hydrogen sulfide in organism and in gastrointestinal tract.

According to the studies cited above, we can conclude that H_2_S as a gaseous mediator plays an important role in many physiological aspects in human body, especially within particular parts of digestive system (Figure 5). In this review article, we focused on potent involvement of the molecule in gastroprotection and maintenance of gastric mucosal integrity.

H_2_S takes part in natural prevention against many disorders of the digestive system but activity of this molecule in GI tract depends on the concentration of this gaseous mediator in particular tissues. Thus, it is very important to improve methods which help to determine precisely the threshold value of H_2_S in tissues.

To summarize, recently published data cited in this article, demonstrated that H_2_S serve as an important physiological molecules within GI tract. H_2_S and NO activity is connected with complex mechanisms (Table 1). These molecules maintain the integrity of gastric mucosa and exert gastroprotection by reducing lesion area caused by various damaging factors within GI tract. Although to clearly determine the protective role of these molecules it is necessary to conduct more studies concerning these aspects and corresponding to already present data.

**Table 1:** Nitric oxide and hydrogen sulfide biochemistry and physiology.

	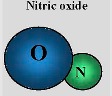	Reference	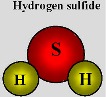	Reference
**Physiological concentrations**				
**serum**	1 nM	[132]	30–100 μM	
**brain/tissue**	100–250 nM	[133]	50–160 μM	[106]
**toxic**	0.5 µM	[134]	250 μM	
**Biochemical properties**				
**Half-life**	Seconds—minutes	[135]	Seconds	[135]
**Physiological forms**	NO exists as a free radical	[136]	20% exist as H_2_S, 80% as HS^−^, trace amounts of S^2−^	[137]
**Crosstalk interaction on catalyzing enzymes**				
	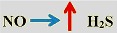		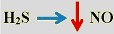	
	NO donor increases the expression and activity of CSE in cultured aortic smooth muscle cells (SMCs)	[97]	NaHS **inhibits** iNOS expression and NO production in macrophage cells (RAW264.7)	[138]
	NO cooperates with H2S via activation of guanylyl cyclase and increase of cGMP	[139]	NaHS treatment **reduces** eNOS activity and expression but not nNOS and iNOS in isolated rat aortas	[140]
	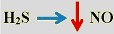		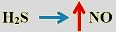	
	NO **does not increase** the expression of H_2_S-generating enzymes and the H_2_S level in endothelial cells.	[141]	NaHS/Na_2_S profoundly **increases** the expression or/and the activity of eNOS	[141,142,143,144]
	H_2_S interacts with NO synthase to transform NO to nitroxyl (HNO) ↓ NO → ↑HNO	[145]	Na_2_S **augmented** NO production in chronically ischemic tissues, by influencing iNOS and nNOS expression and stimulating nitrite reduction to NO via xanthine oxidase (XO) under hypoxic condition	[146]
**Potent mechanisms of gastroprotection**				
**I/R injury**	↑ gastric blood flow ↓ lipid peroxidation ↓ free radicals	[147]	↓ plasma level of IL-1β and TNF-α mRNA expression	[114]
**WRS injury**	↓ lipid peroxidation ↑ SOD activity ↑ GSH concentration	[58]	↓ acid output, ↑ gastric juice pH and mucin concentration, ↑GSH, CAT and SOD enzymes activities	[148]
			↓ lipid peroxidation products	[110]
**Ethanol injury**	↓ free radicals ↑prostaglandins production	[149]	Involvement of K_ATP_ channels, capsaicin-sensitive nerve fibers and TRPV1 receptors	[2]
**Gastric ulcers healing**				
	NO inhibits oxidative stress leading to acceleration of chronic gastric ulcers healing	[150]	Beneficial effect is not dependent on NO synthesis and do not occur through activation of ATP-sensitive K^+^ channels	[90]
